# Transcriptional Regulation of Telomerase Reverse Transcriptase (TERT) by MYC

**DOI:** 10.3389/fcell.2017.00001

**Published:** 2017-01-26

**Authors:** Ekta Khattar, Vinay Tergaonkar

**Affiliations:** ^1^Laboratory of NFκB Signaling, Institute of Molecular and Cell Biology, A*STARSingapore, Singapore; ^2^Department of Biochemistry, Yong Loo Lin School of Medicine, National University of SingaporeSingapore, Singapore; ^3^Centre for Cancer Biology, University of South Australia and SA PathologyAdelaide, SA, Australia

**Keywords:** TERT, Myc, telomerase, transcription, cancer

## Abstract

Telomerase elongates telomeres and is crucial for maintaining genomic stability. While stem cells and cancer cells display high telomerase activity, normal somatic cells lack telomerase activity primarily due to transcriptional repression of telomerase reverse transcriptase (TERT), the catalytic component of telomerase. Transcription factor binding, chromatin status as well as epigenetic modifications at the TERT promoter regulates TERT transcription. Myc is an important transcriptional regulator of TERT that directly controls its expression by promoter binding and associating with other transcription factors. In this review, we discuss the current understanding of the molecular mechanisms behind regulation of TERT transcription by Myc. We also discuss future perspectives in investigating the regulation of Myc at TERT promoter during cancer development.

## Introduction

Telomerase is a reverse transcriptase that elongates telomeres (Blackburn and Collins, 2011). It is a ribonucleoprotein composed of a catalytic subunit telomerase reverse transcriptase (TERT), an RNA template *Terc* and accessory proteins (Cohen et al., 2007; Venteicher et al., 2009). Telomerase activity is detected in highly proliferating cells like stem cells, immune cells and germ cells (Feng et al., 1995; Avilion et al., 1996; Nakamura et al., 1997). However, most somatic human tissues lack telomerase activity (Aisner et al., 2002; Cong et al., 2002). Absence of telomerase activity results in telomere shortening with successive replication cycles. When telomeres become critically short, the DNA damage response is initiated followed by senescence or cell death (Blackburn et al., 2015). While *Terc* and other accessory proteins are ubiquitously expressed, TERT is transcriptionally downregulated, thereby limiting telomerase activity in somatic cells (Avilion et al., 1996; Heiss et al., 1998; Holzmann et al., 1998; Parfait et al., 2000; Wang and Zhu, 2004; Wang et al., 2009). For cancer cells arising from somatic cells, telomere maintenance becomes imperative in order to acquire immortality. Telomere length is maintained by telomerase in 90% of human cancers, while 10% of cancers utilize an alternative mechanism of telomere lengthening termed ALT (Kim et al., 1994; Bryan et al., 1995, 1997; Shay and Bacchetti, 1997). Besides TERT gene amplification and alternate splicing, TERT promoter regulation represents the key mechanism to regulate telomerase activity (Daniel et al., 2012). Thus, investigating transcriptional regulation of TERT is important for understanding cancer development.

Transcriptional regulation of TERT is extremely complex and not completely understood. Several transcription factors and signaling pathways are known to regulate TERT expression. The human TERT promoter contains binding sites for several transcription factors like Myc, SP1, ER, AP1, ETS, HIFs, and upstream stimulatory factors (USFs) (Kyo et al., 1999, 2000; Takakura et al., 1999, 2005; Wu et al., 1999; Goueli and Janknecht, 2003; Anderson et al., 2006; Xu et al., 2008). Amongst these transcription factors, Myc represents an important regulator of TERT transcription (Wu et al., 1999; Takahashi et al., 2007; Marion et al., 2009). Important insights about the interplay between Myc and TERT have been derived using a mouse model of lymphomagenesis in which lymphoma is specifically driven by Myc (Koh et al., 2015).

In the first part of this review, we discuss the role of Myc in modulating TERT transcription. We then describe how multiple cellular signals impinge on Myc to regulate TERT transcription. Lastly, we present correlation analyses between Myc and TERT in several cancers. In summary, we discuss the way forward to better understand the interplay between Myc and TERT in normal physiology and cancer.

## Direct activation of TERT transcription by Myc

**Figure 1:**
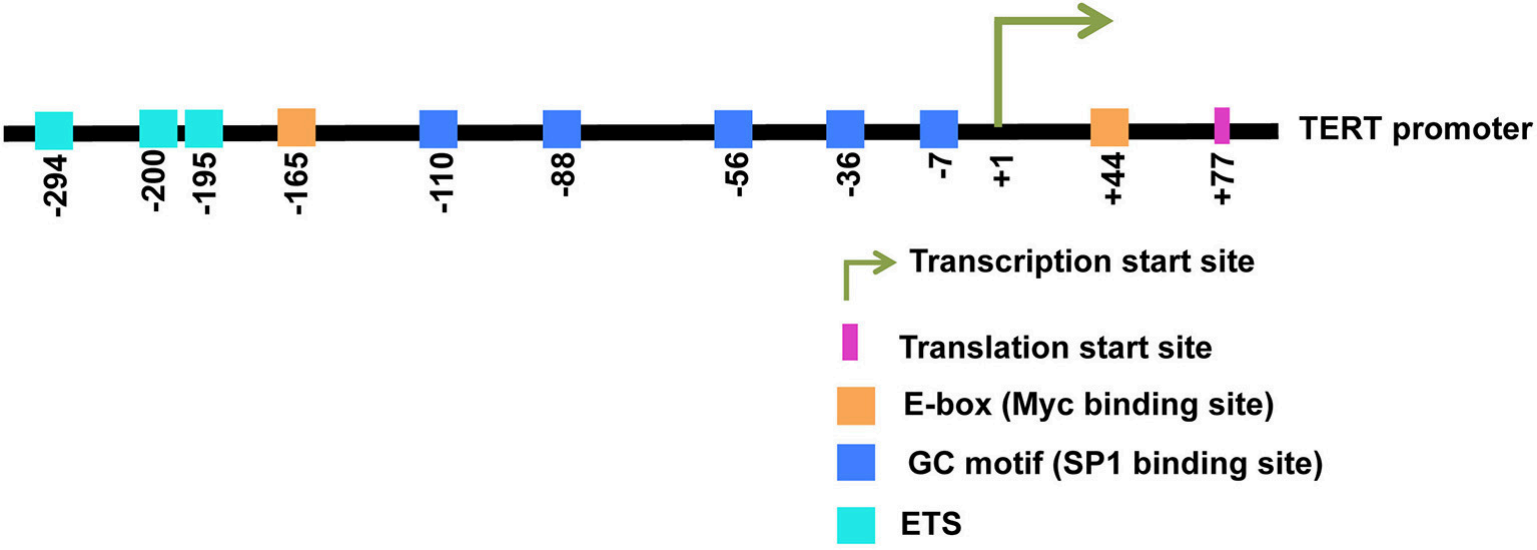
**A schematic representation of the human TERT promoter is shown**. Binding sites for various transcription factors are displayed. Specific nucleotide positions for all binding sites are indicated. +1 indicates the transcription start site and +77 indicates the translation start site.

The TERT core promoter contains two canonical E-box consensus sites (5′-CACGTG-3′) at positions −165 and +44 nucleotide position relative to the transcription start site (TSS) (Horikawa et al., 1999; Takakura et al., 1999; Wick et al., 1999). A schematic representation of the TERT promoter with E-boxes and binding sites for several regulatory transcription factors is presented in Figure 1. E-boxes represent the known binding sites for E-box binding proteins like the Myc/Max/Mad1 superfamily and USFs. Amongst E-box binding proteins, Myc represents an important regulator of TERT transcription. Myc was the first transcription factor to be reported to directly activate TERT transcription in primary fibroblasts as well as in normal epithelial cells (Wu et al., 1999). Myc induces TERT expression independent of cellular proliferation and *de novo* protein synthesis, implying its direct role in regulating TERT transcription. Myc generally activates transcription by recruiting chromatin-modifying complexes like transformation/transcription domain associated protein (TRRAP) and associated histone acetyl transferases (HATs). It can also recruit P-TEFb, which is a kinase that phosphorylates the C-Terminus of RNA polymerase II, thereby helping in promoter clearance. However, the mechanism employed by Myc to activate the TERT promoter is not known (Zhao et al., 2014). The importance of E-box-driven TERT expression has also been investigated in the context of chromatin environment. Mutation of endogenous E-boxes in the TERT promoter can counteract TERT activation by Myc. It is suggested that E-boxes can also function to de-repress the TERT promoter (Zhao et al., 2014).

Myc functions as a heterodimer with Max to activate transcription of genes. The Myc/Max heterodimer is known to activate transcription by inducing topological changes in DNA (Wechsler and Dang, 1992). Structural studies using atomic force microscopy indicate that Myc/Max dimers bind equally and specifically to both E-boxes present in the TERT promoter (Lebel et al., 2007). Although, Myc/Max dimers can oligomerize to form tetramers, the physiological existence of these higher order forms in the context of TERT promoter regulation has not been experimentally verified (Nair and Burley, 2003; Lebel et al., 2007).

## Myc acts as a central signaling factor downstream of major cellular pathways in the regulation of TERT transcription

Myc functions as a downstream effector of several cellular signaling pathways to regulate TERT expression. There are several GC motifs present in the TERT promoter that are recognized by SP1 transcription factors (Kyo et al., 2000). The location of these GC motifs is shown in Figure 1. Transactivation of the TERT promoter by Myc/Max heterodimers is attenuated upon mutation of SP1 sites, suggesting that SP1 and Myc cooperatively function to modulate TERT expression (Kyo et al., 2000). The NF-κB pathway is also reported to regulate TERT transcription. The mammalian NF-κB family comprises of five members, RelA (also termed p65), RelB, c-Rel, NF-κB1 (also termed p50), and NF-κB2 (also termed p52). They associate with each other to form diverse dimeric complexes that regulate numerous target genes via binding to the κB enhancer (Cildir et al., 2016). The canonical NF-κB pathway involves inducible degradation of the IκBs, particularly IκBα, leading to nuclear translocation of various NF-κB complexes, predominantly the p50/RelA dimer (Hayden and Ghosh, 2004). The non-canonical NF-κB pathway activates the RelB/p52 NF-κB complex using a mechanism that does not involve degradation of IκBα, but involves inducible processing of p100 to generate p52 (Sun, 2011). In HTLV-I transformed cells, the viral protein Tax upregulates the canonical NF-κB pathway (Sinha-Datta et al., 2004). This activation of the NF-κB signaling pathway has been indicated to upregulate Myc and SP1 and thus indirectly induce TERT activation. Similarly, an increase in TERT expression by NF-κB-induced activation of Myc/Max has been reported in T lymphocytes activated by PKC θ and in smooth muscle cells stimulated with fibroblast growth factors (Sheng et al., 2006; Bu et al., 2010). The epidermal growth factor (EGF) has also been reported to upregulate TERT transcription via SP1- and Myc/Max-dependent activation of Pyk2/ERK1/2 (Bermudez et al., 2008). In non-small cell lung cancer cell lines, EGF has been reported to activate TERT transcription by inducing direct binding of ETS2 and Myc/Max to the TERT promoter (Hsu et al., 2015). Estrogen has been shown to increase Myc expression and thus indirectly induce TERT transcription, although direct binding of the estrogen receptor (ER) to the TERT promoter has also been demonstrated in ER-positive cancers (Kyo et al., 1999; Cha et al., 2008; Grasselli et al., 2008).

Isocitrate dehydrogenase (IDH) mutations commonly occur in low-grade gliomas (Hartmann et al., 2009; Yan et al., 2009). Mutant IDH1 produces 2-hydroxyglutarate, resulting in altered chromatin modifications, thereby driving gliomagenesis. Recently, it has been shown that mutant IDH1 can reactivate the TERT promoter in human astrocytes by altering the chromatin status and enabling Myc/Max binding (Ohba et al., 2016). This represents a novel mechanism whereby a mutant protein can activate the TERT promoter by modulating its epigenetic status and enabling Myc binding to drive its expression.

The ETS family of transcription factors comprises more than 30 members that contain a characteristic DNA-binding domain known as the erythroblast transformation specific (ETS) domain (Dwyer and Liu, 2010). ETS factors have been shown to contribute to the regulation of telomerase (Maida et al., 2002; Xiao et al., 2003; Goueli and Janknecht, 2004). ETS2 is important for driving TERT gene expression and breast cancer cell proliferation (Dwyer et al., 2007; Xu et al., 2008). Silencing of ETS2 results in a reduction of TERT gene expression leading to increased human breast cancer cell death, while reconstitution with recombinant TERT reverses that effect (Xu et al., 2008). ETS2 has been shown to interact with Myc using co-immunoprecipitation and glutathione S-transferase pull-down assays (Xu et al., 2008). Immunological depletion of ETS2 or mutation of the ETS DNA-binding motif hampers c-Myc binding to the E-box. Further depletion of c-Myc or mutation of the E-box also attenuates ETS2 binding to the ETS DNA-binding motif (Xu et al., 2008). These results suggest that the interaction of ETS2 with c-Myc regulates TERT gene expression, which in turn affects breast cancer cell proliferation. However, this has not been reported in other cancer cell types. Recently, two somatic hotspot mutations were discovered in the TERT promoter that specifically occurred in a subset of cancers (Horn et al., 2013; Huang et al., 2013; Akincilar et al., 2016b). The mutations observed were cytosine to thymine transitions in the TERT promoter and were mutually exclusive (Horn et al., 2013; Huang et al., 2013). These mutations created *de novo* binding sites for ETS transcription factors on the TERT promoter (Bell et al., 2015; Li et al., 2015). GABP, an ETS factor, has been shown to specifically associate with the *de novo* ETS motif on the TERT promoter (Bell et al., 2015). Besides GABP, Li et al. demonstrated that ETS1/2 factors bind cooperatively with NF-kB2 at the mutant TERT promoter during non-canonical NF-kB signaling to drive telomerase reactivation in glioblastomas. These studies suggest that regulation of mutant TERT promoters may be context-dependent in different cancer types and can involve binding of other regulators other than a single ETS factor (Li et al., 2015).

Long-range chromatin interactions mediated by GABP have also been reported to specifically regulate activation of the mutant TERT promoter (Akincilar et al., 2016a). However, cooperation or crosstalk between Myc and GABP to regulate the mutant TERT promoter has not yet been reported.

TERT has also been shown to regulate Myc protein stability by directly interacting with Myc (Koh et al., 2015). This study suggests the existence of a feed-forward loop between Myc and TERT in Myc-driven cancers like lymphoma, wherein Myc upregulates TERT transcription and TERT, in turn, stabilizes Myc protein levels to promote lymphomagenesis. Apart from regulating Myc stability, TERT can also be recruited to Myc target promoters in Myc-driven lymphoma cells probably through association with Myc (Khattar et al., 2016). This represents another novel aspect of the crosstalk between Myc and TERT in cancers.

In contrast to the role of Myc as an activator of TERT transcription, it has also been shown to play a key role in repressing TERT transcription. Inhibition of Myc expression has been reported to increase TERT transcription in normal cells (Zhao et al., 2014). This effect is independent of E-boxes and results in an increase in active histone marks on the TERT promoter.

E2F1 is a direct transcriptional target of Myc and it inhibits TERT transcription by directly associating with the TERT promoter (Crowe et al., 2001; Elliott et al., 2008; Lacerte et al., 2008; Zhang et al., 2012). This exemplifies the dual nature of Myc in regulating TERT transcription. This may also represent another mechanism operating in normal cells to control Myc-dependent oncogenic signals.

A switch from Myc/Max to Max/Mad1 complexes has been shown to repress TERT transcription (Xu et al., 2001). TERT transcription is repressed when stem cells differentiate to specific lineages. The Myc/Max to Max/Mad1 switch is reported to arise during differentiation and has been proposed as one of the mechanisms of repressing TERT transcription.

Peroxisome proliferator-activated receptor γ (PPARγ) ligands inhibit TERT mRNA expression in colon cancer cells via modulation of the Myc/Max/Mad network (Toaldo et al., 2010). PPARγ ligands reduce the levels of Myc and TERT and simultaneously upregulate Mad1 levels, thereby inducing a switch from Myc/Max to Mad1/Max complexes on the TERT promoter. However, the exact molecular mechanism behind the transcriptional changes in Myc and Mad1 levels induced by PPARγ ligands has not been characterized.

## Correlation between Myc and TERT expression in cancers

Alterations in Myc expression are commonly observed during cancer initiation and progression. They occur via chromosomal translocations, amplifications and gene mutations (Dang, 2012). Telomerase reconstitution by reactivating TERT expression is known to occur in cancers by various mechanisms that involve oncogenic transcription factors, gene amplification, promoter mutations, crosstalk with oncogenic pathways or epigenetic regulation (Akincilar et al., 2016b; Li and Tergaonkar, 2016; Li et al., 2016; Tergaonkar, 2016). The correlation between Myc and TERT mRNA expression has been extensively investigated in cancers for two reasons. The first being that simultaneous deregulation of Myc and TERT is a frequent genetic event occurring in several cancers. And secondly, TERT is a transcriptional target of Myc.

Myc expression is usually measured at the protein level using immunohistochemistry. For measurement of TERT expression, some studies use mRNA expression, while others measure telomerase enzyme activity since TERT mRNA levels positively correlate with telomerase activity (Armstrong et al., 2000; Kirkpatrick et al., 2003a). Table 1 lists all the studies that report an association between Myc and TERT expression. In most cancers, high expression of Myc and TERT (measured as mRNA expression or telomerase activity) occur together, suggesting positive correlation. The breast cancer and hepatocellular cancer studies listed in Table 1 show no correlation between Myc and TERT expression. This suggests that there may be additional, as yet undiscovered mechanisms operating for regulation of TERT expression through Myc- dependent or independent means in these cancer types. The reported correlation studies should be interpreted with caution because of the small sample sizes. Further, TERT expression and telomerase activity measurement is not standardized and can therefore vary with different protocols.

**Table 1:** **This table summarizes correlation analyses between: (a) Myc and TERT expression, (b) Myc expression and telomerase activity in various types of cancers**.

**Cancer type (number of studies)**	**Correlation between TERT and Myc mRNA expression^a^**	**Correlation between telomerase activity and Myc mRNA expression^b^**	**References**
Breast Cancer (*n* = 4)	No correlation	Not reported	Kirkpatrick et al., 2003b, 2004; Elkak et al., 2005; Bodvarsdottir et al., 2007
Cervical cancer (*n* = 1)	Positive	Not reported	Sagawa et al., 2001
Hepatocellular carcinoma (*n* = 2)	No correlation	Not reported	Chen et al., 2002; Liu et al., 2004
Ovarian cancer (*n* = 1)	No correlation	Positive	Wisman et al., 2003
Non-small cell lung cancer (*n* = 1)	Positive	Not reported	Geng et al., 2003
Lymphoma (*n* = 1)	Positive	Positive	Klapper et al., 2003
Malignant lipomatous tumors (*n* = 1)	Positive	Positive	Schneider-Stock et al., 2003
Colon cancer (*n* = 1)	Positive	Not reported	Georgakopoulos et al., 2013
Gastric cancer (*n* = 1)	Positive	Not reported	Silva et al., 2012
Prostate cancer (*n* = 1)	Positive	Not reported	Latil et al., 2000

*Positive correlation: high Myc expression co-occurs with high TERT mRNA expression or telomerase activity. No correlation: Myc levels and TERT mRNA expression or Myc levels and telomerase activity show no significant association. Not reported: the investigating group did not examine the correlation*.

## Perspectives

Myc and TERT play crucial roles during normal development as well as in carcinogenesis. Therefore, understanding their interplay in both these processes is important. Myc can activate TERT transcription directly or by cooperating with other transcription factors like SP1, ER and ETS. The factors that discriminate Myc-dependent TERT regulation during normal development from cancer are not known and need further investigation. Mutations in the TERT promoter represent the most common non-coding somatic mutations in cancer and it would be interesting to investigate the role of Myc in regulating the mutant TERT promoter. The existing correlation studies between Myc and TERT are not very conclusive and need further analysis.

## Author contributions

EK conceived, designed and wrote the manuscript. EK and VT revised the manuscript.

### Conflict of interest statement

The authors declare that the research was conducted in the absence of any commercial or financial relationships that could be construed as a potential conflict of interest.
